# Evidence both L-type and non-L-type voltage-dependent calcium channels contribute to cerebral artery vasospasm following loss of NO in the rat

**DOI:** 10.1016/j.vph.2010.06.002

**Published:** 2010-09

**Authors:** A.J. McNeish, Francesc Jimenez Altayo, C.J. Garland

**Affiliations:** a Department of Pharmacology, University of Oxford, Mansfield Road Oxford OX1 3QT, United Kingdom; b Department of Pharmacy and Pharmacology, University of Bath, BA2 7AY, United Kingdom

**Keywords:** Cerebral vasospasm, K-channels, T-type calcium channels, Nitric oxide, Endothelial cell dysfunction

## Abstract

We recently found block of NO synthase in rat middle cerebral artery caused spasm, associated with depolarizing oscillations in membrane potential (E_m_) similar in form but faster in frequency (*circa* 1 Hz) to vasomotion. T-type voltage-gated Ca^2+^ channels contribute to cerebral myogenic tone and vasomotion, so we investigated the significance of T-type and other ion channels for membrane potential oscillations underlying arterial spasm. Smooth muscle cell membrane potential (E_m_) and tension were measured simultaneously in rat middle cerebral artery. NO synthase blockade caused temporally coupled depolarizing oscillations in cerebrovascular E_m_ with associated vasoconstriction. Both events were accentuated by block of smooth muscle BK_Ca_. Block of T-type channels or inhibition of Na^+^/K^+^-ATPase abolished the oscillations in E_m_ and reduced vasoconstriction. Oscillations in E_m_ were either attenuated or accentuated by reducing [Ca^2+^]_o_ or block of K_V_, respectively. TRAM-34 attenuated oscillations in both E_m_ and tone, apparently independent of effects against K_Ca_3.1. Thus, rapid depolarizing oscillations in E_m_ and tone observed after endothelial function has been disrupted reflect input from T-type calcium channels in addition to L-type channels, while other depolarizing currents appear to be unimportant. These data suggest that combined block of T and L-type channels may represent an effective approach to reverse cerebral vasospasm.

## Graphical abstract

**Figure:**
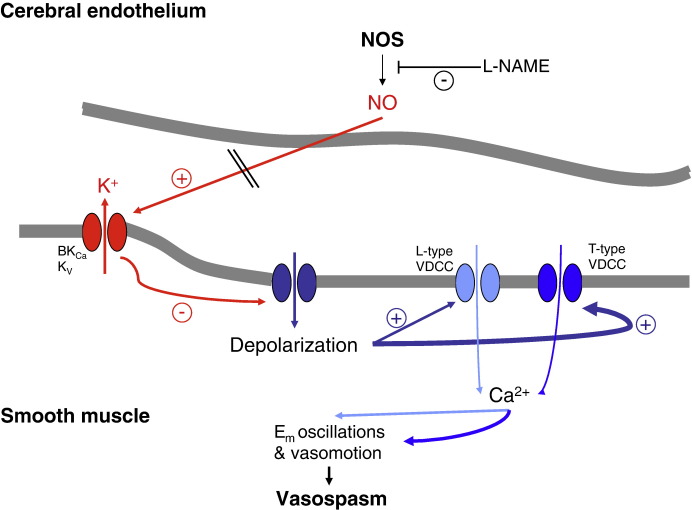

## 1 Introduction

Cerebral arteries operate in a dynamic state of partial constriction (myogenic tone), providing the capacity to constrict or relax in response to changing levels of intraluminal pressure, shear stress and nerve activity. Myogenic tone is an intrinsic property of the smooth muscle, helping maintain constant total cerebral blood flow and adapting blood flow locally to meet metabolic demand. Myogenic constriction is driven primarily by membrane depolarization leading to Ca^2+^ influx (Davis and Hill, 1999; Hill et al., 2001), possibly with a contribution via stretch-activated calcium sensitization (Schubert et al., 2008). Myogenic tone is often superimposed by vasomotion in the form of synchronised oscillations in smooth muscle cell membrane potential (Em), Ca^2+^ and tension. Although the physiological function of vasomotion in general is unclear, it may help to maintain a constant blood supply in many tissues, including the brain (Gustafsson, 1993; Haddock and Hill, 2005).

One key influence of basal NO release in the middle cerebral artery appears to be suppression of both myogenic tone (Golding et al., 2001; Peng et al., 1998; Zimmermann et al., 1997) and vasomotion (Dirnagl et al., 1993; Haddock and Hill, 2002). This influence appears largely due to activation of BK_Ca_ (Brayden and Nelson, 1992; Mandala et al., 2007; Yuill et al., 2010). So block of NO generation and/or BK_Ca_ provides a means to mimic an aspect of endothelial dysfunction that is an early feature of cardiovascular disease, including disease conditions that predispose to vasospasm (Jewell et al., 2004; Vanhoutte et al., 2009). These conditions will also mimic the loss of NO observed after subarachnoid haemorrhage, where scavenging of NO by haemoglobin (Martin et al., 1985) causes profound vasospasm (Toda et al., 1991). Significantly, enhanced vasomotion (or vasospasm) can lead to a reduction of cerebral capillary blood flow and thus compromise of neuronal function (Biswal and Hudetz, 1996; Pluta, 2005).

Several mechanisms, including the rho-kinase pathway, can contribute to the development and maintenance of constriction in smooth muscle, alongside calcium entry. However, in many vascular beds, including the cerebral vasculature changes in smooth muscle intracellular Ca^2+^ ([Ca^2+^]_i_) concentration is critical for myogenic tone and vasomotion (Haddock and Hill, 2002, 2005; Yuill et al., 2010). [Ca^2+^]_i_ increase involves release from intracellular stores and entry from the extracellular space via voltage-gated Ca^2+^ channels and non-selective cation channels, such as transient receptor potential channels (TRPC). Ca^2+^ influx through voltage-gated Ca^2+^ channels (VGCC) leads to global increases in smooth muscle cell [Ca^2+^]_i_ and constriction, and high voltage activated (L-type) Ca^2+^ channels appear central in this sequence (McCarron et al., 1997; Moosmang et al., 2003; Nelson et al., 1990). These channels are expressed widely in vascular smooth muscle and their open probability increases over a physiologically relevant range (circa −50 to −30 mV) (Lacinova, 2005; Smirnov and Aaronson, 1992). Low voltage activated or T-type Ca^2+^ channels are also expressed in vascular smooth muscle of resistance arteries (Braunstein et al., 2008; Clozel et al., 1997; Kuo et al., 2008; Navarro-Gonzalez et al., 2009; Perez-Reyes, 2003). But although they are normally active in the range *circa* −60 to −40 mV, the characteristic rapid inactivation of these channels argues against a significant role at physiologically relevant membrane potentials in the vasculature. Despite this, they have been implicated in the maintenance of vascular tone in a variety of arteries, including rat cremaster (VanBavel et al., 2002), rat basilar (Navarro-Gonzalez et al., 2009) and middle cerebral (Lam et al., 1998) arteries, and direct measurements have shown high voltage activated but nifedipine-insensitive Ca^2+^ currents, pharmacologically indistinguishable from T-type currents, in both guinea-pig and rat terminal mesenteric arteries (Gustafsson, 1993; Morita et al., 1999; Morita et al., 2002).

We recently reported that middle cerebral arteries develop intense and sustained constriction, associated with a very rapid form of vasomotion, when NOS and/or BK_Ca_ channels were blocked, to mimic endothelial dysfunction. Furthermore, both constriction and vasomotion depended on calcium entry via VGCCs and the oscillations in E_m_ were temporally linked to changes in smooth muscle [Ca^2+^]_i_ (Yuill et al., 2010). The temporally linked oscillations in E_m_, [Ca^2+^]_i_ and tension were similar to the widely described phenomenon of vasomotion, but displayed a much higher frequency (∼ 1 Hz as opposed to ∼ 0.1–0.2 Hz, Yuill et al., 2010). The intense “vasospastic vasomotion” was reversed by inhibition L-type Ca^2+^ channels and clearly involved a complex action of NO that appeared to include stimulation of BK_Ca_ channels and a cGMP-independent closure of VGCCs (Yuill et al., 2010). However, although a central role for VGCC, NO and BK_Ca_ was apparent, the importance of other ionic currents that might contribute to the rapid depolarizing oscillations was unclear.

Thus, the aim of the present study was to characterize the ionic mechanisms responsible for rhythmic oscillations in E_m_ and tension in rat isolated middle cerebral arteries following inhibition of BK_Ca_ channels and NOS. We probed channels that may lead to both depolarization (calcium, sodium and chloride channels) and repolarization (potassium channels). Our data suggest a novel role for both smooth muscle T-type Ca^2+^ channels and several potassium conductances in the both “vasospastic vasomotion” and the underlying maintenance of vasoconstriction.

## 2 Materials and methods

### 2.1 Animals and tissue isolation

Male Wistar rats (200–250 g) were killed by cervical dislocation followed by decapitation, following institutional guidelines for animal welfare and schedule 1 of the Animals (scientific procedures) Act 1986. The brain was removed and immediately placed in ice-cold Krebs solution. Segments of the middle cerebral artery (∼ 2 mm long) were dissected and stored in ice-cold Krebs for use within 30 min, with similar size vessels used in all experimental groups.

### 2.2 Experimental protocols

Segments of middle cerebral artery (internal diameter ∼ 150 μm) were mounted in a Mulvany–Halpern myograph (model 400A, Danish Myotechnology) in Krebs solution containing (mM): NaCl, 118.0, NaCO_3_, 24; KCl, 3.6; MgSO_4_·7H_2_O, 1.2; glucose, 11.0; CaCl_2_, 2.5; gassed with 20% O_2_, 5% CO_2_ and balance N_2_ and maintained at 37 °C. After equilibration for 20 min, vessels were tensioned to 1–1.5 mN (approximates wall tension at 60 mmHg). Smooth muscle tension was recorded with an isometric force transducer and Powerlab software (ADI, Australia). Vessel viability was assessed by addition of exogenous K^+^ (15–55 mM, total K^+^ concentration); only vessels developing tension of ≥ 3 mN were used, following this Endothelial cell viability was assessed by the ability of the protease activated receptor 2 activating peptide; SLIGRL (20 μM) (Alexander et al., 2008) to relax U46619 induced tone (100 nM) by ≥ 75%, vessels with less relaxation were discarded. In some experiments, endothelial cells were removed by gently rubbing the luminal surface with a human hair; subsequent relaxation of < 15% to SLIGRL (20 μM) was considered as successful removal and further abrasion often lead to damage of smooth muscle cells.

L-NAME (100 μM), indomethacin (10 μM) and iberiotoxin (100 nM) were added throughout the experiment (to block NO synthase (NOS), cyclooxygenase and BK_Ca_ channels, respectively), unless otherwise stated. In combination, these drugs gave a robust and sustained constriction (similar to vasospasm), and an associated rapid vasomotion. Similar responses were recorded in each case after inhibition of NOS alone, but vasomotion was more variable between preparations. Indomethacin had no effect on oscillations, but was included in the experimental cocktail to minimize any potential for confounding thromboxane signaling after NOS inhibition (Benyo et al., 1998; McNeish and Garland, 2007). Recordings were assessed in the presence of: the T-type (mibefradil 100 nM and NNC 55-0396 300 nM) and L-type (nifedipine 1 μM) Ca^2+^channel blockers, the K_Ca_ channel blockers, apamin (K_Ca_2.3 (SK_Ca_), 50 nM), TRAM-34 (K_Ca_3.1 (IK_Ca_), 1 μM), iberiotoxin (BK_Ca_, K_Ca_1.1, 100 nM) and charybdotoxin (K_Ca_3.1, B_Ca_, 100 nM), the K_IR_ channel inhibitors BaCl_2_ (30 μM) and CsCl_2_ (10 mM), the Na^+^/K^+^-ATPase inhibitor, ouabain (1 μM) and the voltage-gated K^+^ channel inhibitor, 4-aminopyridine (4-AP, 3 mM). Papaverine (150 μM) was added at the end of each experiment to assess overall tone. All blocking drugs were incubated for at least 20 min before data was recorded to ensure maximal effect. In most experiments smooth muscle membrane potential (E_m_) and tension were measured simultaneously as previously described, using glass microelectrodes (filled with 2 M KCl; tip resistance, 80–120 MΩ) to measure E_m_ (Garland and McPherson, 1992).

### 2.3 Data analysis and statistical procedures

Results are expressed as the mean ± s.e. mean of *n* animals. Tension values are given in mN (always per 2 mm segment) and E_m_ as mV. During the vasospastic vasomotion E_m_ is expressed as the mean E_m_ over a random 10 s period of the rapid vasomotion where possible we have also reported the size of the depolarizing oscillations in mV. Vasodilatation is expressed as percentage reduction of the total vascular tone (myogenic tone plus vasoconstrictor response induced by either U46619 or the combination of L-NAME and iberiotoxin, as appropriate), quantified by relaxation with papaverine (150 μM). Graphs were drawn and comparisons made using either Student's t-test, or one-way ANOVA with Tukey's post-hoc test using Prism software (Graphpad, USA). P ≤ 0.05 was considered significant.

### 2.4 Drugs, chemicals, reagents and other materials

Exogenous K^+^ was added as an isotonic physiological salt solution in which all the NaCl was replaced with an equivalent amount of KCl. Concentrations of K^+^ used are expressed as final bath concentration. L-NAME (N^G^-nitro-l-arginine methyl ester), indomethacin, mibefradil, NNC 55-0396, nifedipine, barium chloride, cesium chloride, ouabain, 4-aminopyridine and papaverine were all obtained from Sigma (Poole, U.K.). U46619 (9,11-Dideoxy-11α,9α-epoxymethanoprostaglandin F2α) was from Calbiochem (UK). Apamin, charybdotoxin, iberiotoxin and tetrodotoxin from Latoxan (Valence, France). SLIGRL (serine–leucine–isoleucine–glycine–arginine–leucine–NH2) from Auspep (Parkville, Australia). TRAM-34 was a generous gift from Dr H. Wulff (University of California, Davis). All stock solutions were dissolved in distilled water except SLIGRL and charybdotoxin, dissolved in 0.9% NaCl, U46619 and TRAM-34, dissolved in dimethylsulfoxide (DMSO), nifedipine, dissolved in ethanol, and indomethacin which was dissolved in Na_2_CO_3_ (2%); vehicle controls were performed for drugs dissolved in DMSO ethanol and Na_2_CO_3_. All nomenclature conforms to the BJP guide to receptors and ion channels (Alexander et al., 2008).

## 3 Results

### 3.1 Effect of inhibiting Nitric oxide synthase and BK_Ca_ on E_m_ and tension.

Rat middle cerebral arteries developed spontaneous myogenic tone equivalent to 1.3 ± 0.1 mN (≈ 15% of maximum tension, with 55 mmol/L KCl, n = 40) with a resting membrane potential (E_m_) of −42.1 ± 0.9 mV (n = 40). Addition of the NOS inhibitor, L-NAME (100 μM), and the cyclooxygenase inhibitor, indomethacin (10 μM) tended to evoke smooth muscle cell depolarization (E_m_ −39.2 ± 1.0 mV, n = 38) and constriction (4.6 ± 0.2 mN, n = 40; *P* < 0.05). In all vessels, the depolarization developed into ongoing oscillations (amplitude 7 ± 0.9 mV) followed by equivalent changes in tone (amplitude 0.12 ± 0.01 mN). We have previously reported similar observations in the rat middle cerebral artery following NOS inhibition alone (Yuill et al., 2010). With L-NAME present, stimulation of the endothelium with 20 μM SLIGRL evoked hyperpolarization of 20.9 ± 1.7 mV (n = 31) associated with 77.6 ± 3.6% relaxation (n = 37).

**Fig. 1:**
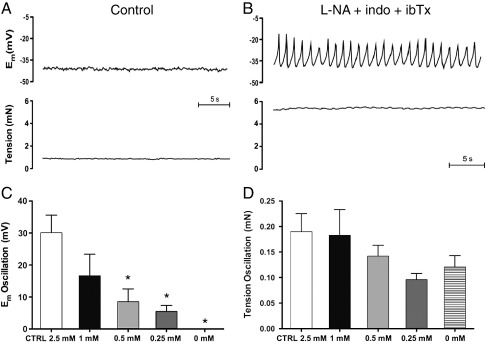
Original traces showing simultaneous recordings of membrane potential (upper panels) and tension (lower panels) in rat middle cerebral arteries under control resting conditions (A) or in the presence of L-NAME (100 μM), indomethacin (10 μM) and iberiotoxin (100 mN; B). Under control conditions, membrane potential and tension are relatively stable. In the presence of L-NAME, indomethacin and iberiotoxin, the smooth muscle cells depolarized and developed regular depolarizing oscillations, which were associated with constriction and oscillations in tension; the peaks in membrane potential immediately preceded peaks in tension. Decreasing extracellular calcium (from 2.5 to 0 mM Ca^2+^) caused depolarization and relaxation. Average data are shown in (C and D) showing the oscillation amplitude in membrane potential (C) and tension (D) in control vessels (2.5 mM Ca^2+^) and in the presence of 1, 0.5, 0.25 and 0 mM Ca^2+^. Data expressed as means ± S.E.M. * *P* ≤ 0.05 indicates a significant difference from control by one-way ANOVA with Tukey's post-hoc test, n = 3–7.

In the presence of L-NAME and indomethacin, the BK_Ca_ channel inhibitor, iberiotoxin (100 nM), evoked further depolarization (to E_m_ −35.7 ± 1.1 mV, n = 40; *P* < 0.05) and constriction (to 5.7 ± 0.2 mN, n = 41; *P* < 0.05), associated with a marked increase in the amplitude of oscillations in E_m_ temporally linked to tension (Fig. 1B). The oscillations in E_m_ and tension had a frequency of 0.84 ± 0.02 Hz and 0.80 ± 0.05 Hz and amplitude of 22.6 ± 1.3 mV and 0.19 ± 0.02 mN, respectively (n = 41). All subsequent experiments were performed in the presence of L-NAME, indomethacin and iberiotoxin unless stated. Removal of the endothelium abolished SLIGRL-mediated relaxation (20 μM), but failed to affect oscillations in E_m_ (frequency of 0.76 ± 0.10 Hz; amplitude of 19.4 ± 4.3 mV) and tension (frequency of 0.66 ± 0.10 Hz; amplitude of 0.16 ± 0.05 mN, n = 3).

### 3.2 [Ca^2+^]_o_ but not Na^+^ or Cl^−^ currents modify oscillations in membrane potential

The voltage-dependent Na^+^ channel blocker, tetrodotoxin (1 μM) did not modify either the frequency or amplitude of oscillations in E_m_ (control: frequency of 0.84 ± 0.03 Hz, amplitude of 16.1 ± 1.4 mV; tetrodotoxin: frequency of 0.90 ± 0.02 Hz, amplitude of 15.4 ± 2.7 mV, n = 3) and tension (control: frequency of 0.73 ± 0.11 Hz, amplitude of 0.13 ± 0.01 mN; tetrodotoxin: frequency of 0.67 ± 0.14 Hz, amplitude of 0.12 ± 0.01 mN, n = 3).

To investigate if the oscillations were dependent upon Ca^2+^ influx, we decreased extracellular Ca^2+^ in steps from 2.5 to 0 mM. Simultaneous measurements of changes in E_m_ and tension showed that decreasing Ca^2+^ evoked depolarization (from −37.0 ± 2.1 to −26.8 ± 2.4 mV, n = 7; *P* < 0.05) and relaxation (from 5.4 ± 0.6 to 0.3 ± 0.1 mN, n = 7; *P* < 0.05). Under these conditions, the amplitude of oscillations in E_m_ and tension was diminished (Fig. 1C and D). The calcium-dependent Cl^−^ channel inhibitor, DIDS (150 μM), did not alter spontaneous oscillations in E_m_ or tension (data not shown) nor did it modify E_m_ (control: −44.4 ± 3.0 mV; DIDS: −49.2 ± 3.9 mV, n = 5) or tension (control: 4.7 ± 0.6 mN; DIDS: 3.6 ± 0.5 mN, n = 5).

### 3.3 Effect of Ca^2+^channel blockers on oscillations in E_m_ and tension

**Fig. 2:**
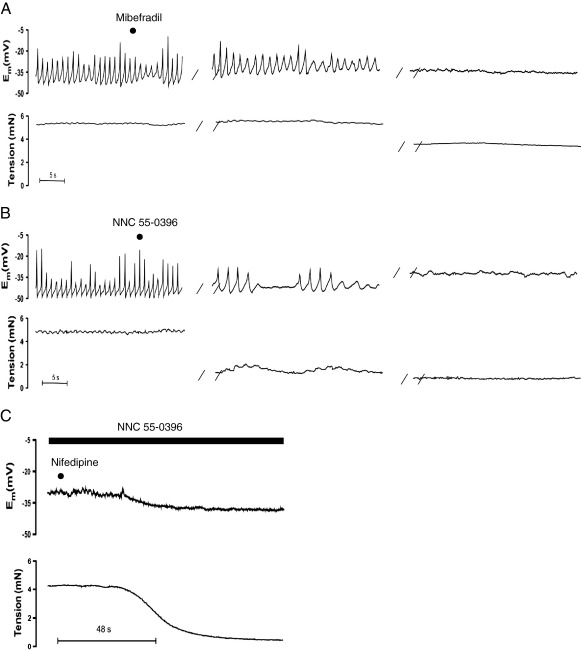
Original traces showing the effect of either (A) the T-type Ca^2+^ channel blocker, mibefradil (100 nM), (B) the more selective T-type Ca^2+^ channel antagonist, NNC 55-0396 (300 nM) or (C) the combined application of NNC 55-0396 (300 nM) and the L-type Ca^2+^ channel blocker, nifedipine (1 μM), on simultaneous recordings of membrane potential (upper panels) and tension (lower panels). Both mibefradil and NNC 55-0396 caused relaxation and abolished oscillations in membrane potential and tension; combined application of NNC 55-0396 and nifedipine caused greater hyperpolarization and relaxation than NNC-550396 alone, n = 4–6. Parallel lines (//) indicate a time break between same recordings from a single vessel the first time break shows the response at approximately 5 min post addition of drug the second time break corresponds to the maximum response which is approximately 15 min following addition.

The T-type selective Ca^2+^ channel blocker, mibefradil (100 nM) abolished oscillations in E_m_ (Fig. 2A), significantly reduced oscillations in tension (control: frequency of 0.79 ± 0.07 Hz, amplitude of 0.16 ± 0.04 mN; mibefradil: frequency of 0.09 ± 0.05 Hz, amplitude of 0.04 ± 0.02 mN, n = 5; *P* < 0.05) and evoked relaxation (control: 5.1 ± 0.7 mN; mibefradil: 3.5 ± 0.6 mN, n = 5) and depolarization (E_m_ control: −36.1 ± 1.3 mV; E_m_ mibefradil: −30.4 ± 2.5 mV, n = 5). The more selective T-type Ca^2+^ channel antagonist, NNC 55-0396 (300 nM) also significantly reduced oscillations in E_m_ (Fig. 2B) and in tension (control: frequency of 0.79 ± 0.09 Hz, amplitude of 0.17 ± 0.03 mN; NNC 55-0396: frequency of 0.20 ± 0.07 Hz, amplitude of 0.07 ± 0.02 mN, n = 6; *P* < 0.05) and evoked relaxation (control: 5.5 ± 0.5 mN; NNC 55-0396: 3.0 ± 0.5 mN, n = 6; *P* < 0.05). However, NNC 55-0396 did not significantly modify mean E_m_ (E_m_ control: −36.2 ± 3.2 mV; E_m_ NNC 55-0396: −31.9 ± 3.2 mV, n = 6). Further addition of the L-type voltage-gated Ca^2+^ channel inhibitor, nifedipine (1 μM), relaxed (control: 5.8 ± 0.7 mN; NNC 55-0396 3.5 ± 0.6 mN; NNC 55-0396 + nifedipine: 0.5 ± 0.1 mN, n = 4; *P* < 0.05) and hyperpolarized (E_m_ control: −33.0 ± 3.7 mV; E_m_ NNC 55-0396: −30.0 ± 4.6; Em NNC 55-0396 + nifedipine: −36.7 ± 3.4 mV, n = 4; Fig. 2C). We also assessed the effect of NNC 55-0396 against middle cerebral artery basal myogenic tone in the absence of inhibitors. NNC 55-0396 (300 nM) did not significantly affect E_m_ (E_m_ control: −51.6 ± 2.47; E_m_ NNC 55-0396: −48.9 ± 2.0 mV, n = 5) or relax myogenic tone (control: 1.04 ± 0.18; NNC 55-0396: 0.69 ± 0.09 mN) under these conditions. The additional presence of nifedipine (1 μM) also failed to evoke hyperpolarization (E_m_ NNC 55-0396+ nifedipine: −51.5 ± 2.8 mV) but did cause significant relaxation (NNC 55-0396+ nifedipine: 0.44 ± 0.04 mN, n = 4, P < 0.05). We have previously demonstrated similar relaxation of basal myogenic tone with nifedipine alone (Yuill et al., 2010).

### 3.4 Involvement of K_Ca_3.1(IK_Ca_) and K_Ca_2.3(SK_Ca_) channels

**Fig. 3:**
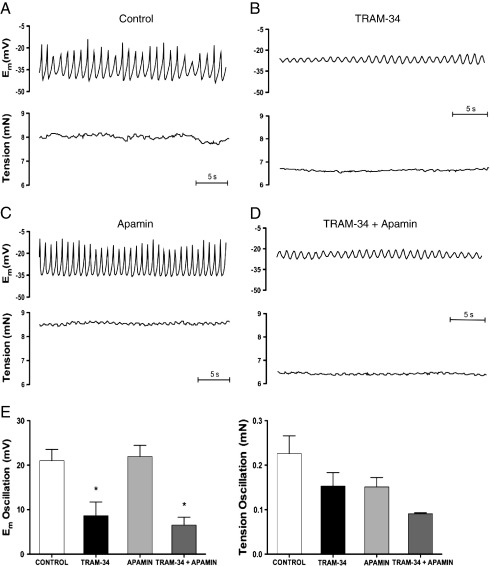
Original traces showing control conditions (A), the effect of the K_Ca_3.1 channel blocker, TRAM-34 (1 μM; B), the K_Ca_2.3 channel blocker, apamin (50 nM; C) or the combined application of TRAM-34 and apamin (D) on simultaneous recordings of membrane potential (upper panels) and tension (lower panels). Average data for oscillation amplitude in membrane potential (left panel) and tension (right panel) in control vessels and in the presence of TRAM-34, apamin and TRAM-34 + apamin are also shown (E). Data are expressed as means ± S.E.M. * *P* ≤ 0.05 indicates a significant difference from control by one-way ANOVA with Tukey's post-hoc test, n = 3–9.

**Fig. 4:**
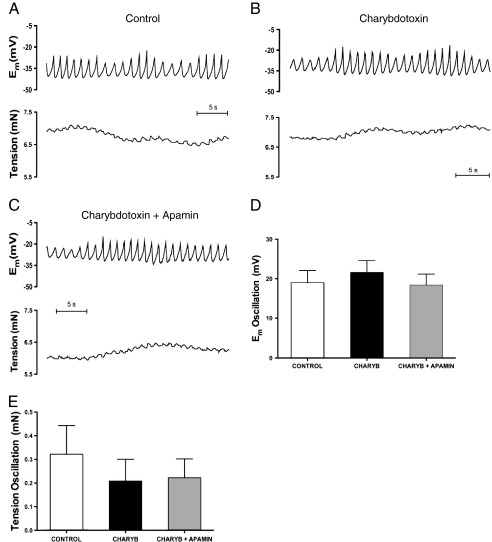
Original traces showing control conditions (A) and the effect of either the BK_Ca_ and K_Ca_3.1 channel blocker, charybdotoxin (100 nM; B) or the combined application of charybdotoxin (100 nM) and the K_Ca_2.3 channel blocker, apamin (50 nM; C) on simultaneous recordings of membrane potential (upper panels) and tension (lower panels). Average data are shown for the oscillation amplitude in membrane potential (D) and tension (E) in control vessels and in the presence of charybdotoxin and charybdotoxin + apamin. Data expressed as means ± S.E.M. n = 3–4.

Addition of the K_Ca_3.1 channel blocker, TRAM-34 (1 μM) markedly decreased the amplitude of oscillations in E_m_ and tension (Fig. 3B) followed by small relaxation (control: 6.5 ± 0.4 mN; TRAM-34: 5.3 ± 0.2 mN, n = 5; *P* < 0.05). TRAM-34 did not significantly reduce E_m_, (E_m_ control: −33.9 ± 2.6 mV; E_m_ TRAM-34: −28.7 ± 2.3 mV, n = 4). The effect of TRAM-34 on the amplitude of oscillations in E_m_ (control: 19.4 ± 4.3 mV; TRAM-34: 3.0 ± 1.6 mV, n = 3; *P* < 0.05) and tension (control: 0.16 ± 0.05 mN; TRAM-34: 0.04 ± 0.02 mN, n = 3) was similar after endothelium removal. The oscillations in E_m_ and tension were not modified by the K_Ca_2.3 blocker apamin (50 nM), either alone (Fig. 3C) or in the additional presence of TRAM-34 (Fig. 3D). Likewise, charybdotoxin (100 nM) alone (Fig. 4B) or with apamin (Fig. 4C) did not affect either oscillations in E_m_ and tension (Fig. 4D and E), or mean tension and E_m_.

### 3.5 Involvement of K_IR_ channels, voltage-gated K^+^ channels and the Na^+^/K^+^-ATPase

**Fig. 5:**
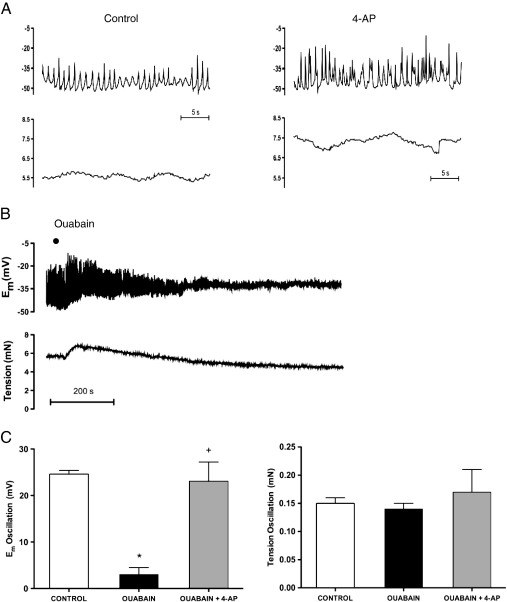
Original traces showing (A) the effect of the voltage-gated K^+^channel inhibitor, 4-AP (3 mM) and (B) the effect of the Na^+^/K^+^-ATPase inhibitor, ouabain (1 μM) on simultaneous recordings of membrane potential (upper panels) and tension (lower panels). Average data are shown in (C) showing the oscillation amplitude in membrane potential (left panel) and tension (right panel) in control vessels and in the presence of ouabain and ouabain + 4-AP. Data expressed as means ± S.E.M. */^**+**^*P* ≤ 0.05 indicates a significant difference from either control or from ouabain alone, respectively, using one-way ANOVA with Tukey's post-hoc test, n = 3.

Inhibition of K_IR_ channels with BaCl_2_ (30 μM) did not affect the amplitude of oscillations in E_m_ (control: 31.1 ± 4.6 mV; BaCl_2_: 30.9 ± 6.1 mV, n = 5) or tension (control: 0.20 ± 0.03 mN; Ba^2+^: 0.19 ± 0.03 mV, n = 5). However, another inhibitor of K_IR_, CsCl (10 mM) increased the amplitude of oscillations in E_m_ (control: 30.8 ± 2.0 mV; CsCl: 40.4 ± 3.4 mV, n = 5; *P* < 0.05), but not in tension (control: 0.20 ± 0.02 mN; CsCl: 0.19 ± 0.02 mV, n = 5), while 4-aminopyridine (4-AP; 3 mM to block K_v_) increased the amplitude of oscillations in both E_m_ (control: 17.75 ± 2.07; 4-AP: 30.62 ± 7.33 mV, n = 3) and tension (control: 0.17 ± 0.01; 4-AP: 0.25 ± 0.07 mN; n = 3, Fig. 5A). The Na^+^/K^+^-ATPase inhibitor, ouabain (1 μM) evoked relaxation (control: 5.8 ± 0.3 mN; ouabain: 4.6 ± 0.2 mN, n = 3; *P* < 0.05) but without significantly reducing E_m_ (control: −42.2 ± 1.2 mV; ouabain: −38.4 ± 1.6 mV, n = 3). However, ouabain did reduce both the amplitude (Fig. 5B and C) and frequency (control: 0.97 ± 0.11 Hz; ouabain: 0.31 ± 0.16 Hz, n = 3; *P* < 0.05) of oscillations in E_m_, without altering oscillations in tension (Fig. 5C and frequency control: 1.30 ± 0.22 Hz; plus ouabain: 0.92 ± 0.22 Hz, n = 3). The subsequent addition of 4-AP in the presence of ouabain caused depolarization (ouabain: −38.4 ± 1.6 mV; ouabain +4-AP: −34.6 ± 3.3 mV, n = 3; P < 0.05), and increased oscillations in Em (Fig. 5C) and increased tension overall (ouabain: 4.6 ± 0.2 mN; ouabain + 4-AP: 5.5 ± 0.2 mN, n = 3; P < 0.05).

## 4 Discussion

This study provides the first demonstration, that rhythmic oscillations in membrane potential and tension as well as the associated spasm in rat isolated middle cerebral arteries following inhibition of BK_Ca_ channels and/or NOS reflect Ca^2+^ influx via T-type Ca^2+^ channels, in addition to L-type Ca^2+^ channels. These data extend our previous observation that NOS inhibition leads to L-type Ca^2+^ channel opening and arterial spasm, characterized by sustained constriction and superimposed by rapid vasomotion (Yuill et al., 2010). Vasomotion was of much higher frequency than previously recorded in other vascular beds, and as such we refer to it as ‘vasospastic’ vasomotion. We also provide evidence for modulation of both the Ca^2+^ dependent vasomotion and constriction through Na/K ATPase and a K^+^ conductance.

Consistent with previous work, our data suggest that calcium influx underlies rhythmic oscillations in the constricted rat middle cerebral artery, and that oscillations in membrane potential and tension are linked to oscillations in intracellular [Ca^2+^]_i_ as well as spasm in both middle cerebral and basilar arteries (Haddock and Hill, 2002; Navarro-Gonzalez et al., 2009; Yuill et al., 2010). Reducing extracellular calcium diminished the amplitude of oscillations, led to relaxation and paradoxically depolarized the membrane. The relaxation presumably reflected the reduction in peak E_m_ associated with the reduced amplitude of oscillations in E_m_. Some small oscillations in tension did persist and may reflect vasomotion-independent intracellular calcium release, as reported in some arterial beds: for review see Haddock and Hill (2005). As oscillations were insensitive to the calcium-dependent Cl^−^ channel inhibitor, DIDS and the voltage-dependent Na^+^ channel blocking agent, tetrodotoxin they certainly appeared to be mediated exclusively by calcium conductance. These data contrast with the basilar artery, where inhibition of Cl^−^ channels in this larger artery abolished calcium-dependent oscillations, leading to hyperpolarization and relaxation (Haddock and Hill, 2002) and parenchymal arterioles, where calcium-dependent oscillations were blocked with tetrodotoxin (Filosa et al., 2004). However, our data are consistent with human pial arteries, where tetrodotoxin was also without effect against oscillations in diameter (Gokina et al., 1996). In the present study, oscillations in muscle membrane potential were also resistant to direct damage of the endothelium, suggesting that this monolayer may not influence rapid vasomotion associated with arterial spasm.

In the rat middle cerebral artery, opening L-type Ca^2+^ channels is essential for vasoconstriction and vasomotion to develop, because inhibition of these channels abolishes vasomotion and fully reverses tone (Yuill et al., 2010). Surprisingly, in the present study under similar vasospastic conditions, oscillations in E_m_, and the associated oscillations in tension were abolished and followed by relaxation after calcium influx through T-type Ca^2+^ channels was blocked with mibefradil (at a concentration selective for block of T-type Ca^2+^ channels; 100 nM) or with a T-type Ca^2+^ channel selective, non-hydrolysable analogue of mibefradil, NNC 55-0396, (Huang et al., 2004). Block of oscillations with the putative T-type Ca^2+^ channel blockers was not associated with a net hyperpolarization and complete relaxation, contrasting with the L-Type Ca^2+^ channel blocker nifedipine. So the effect of mibefradil or NNC 55-0396 is unlikely to reflect a non-specific effect against L-type channels. Furthermore, although mibefradil apparently reduced blood pressure and myogenic tone by an action on L-type calcium channels (Moosmang et al., 2006), the lower concentration of mibefradil used in the present study is relatively specific against T-type Ca^2+^ channels. In fact, mibefradil seems only to inhibit the L-type Ca^2+^ channels after tissue metabolism (Wu et al., 2000). The non-hydrolysable analogue of mibefradil NNC 55-0396 is selective for T-type Ca^2+^ channels, with no reported block of L-type Ca^2+^ channels even in concentrations as high as 100 μM (Huang et al., 2004). Mibefradil has also been reported to block both Cl^−^ (Bernd et al., 1997) and Na^+^ (Eller et al., 2000; Guatimosim et al., 2001) channels, but again with much higher concentrations (μM) than employed in the current study. Furthermore, the fact that blockers such as DIDs and TTX had no effect against vasospastic vasomotion makes an action of mibefradil against these channels extremely unlikely. Therefore, the differential effect of mibefradil and NNC-0396 compared to the selective blocker L-type Ca^2+^ channel blocker nifedipine indicates a critical role for T-type Ca^2+^ channels in vasospastic vasomotion. Further, these channels contribute significantly to the overall constriction in the middle cerebral artery.

Vasospastic vasomotion only developed once the smooth muscle cells depolarized to *circa* −40 mV, so it may be that T-type channels involved in vasomotion have gating properties similar to high voltage activated Ca^2+^ channels. This is surprising, as by definition T-type Ca^2+^ channels activate at low potentials and then quickly inactivate (Lacinova, 2005). However, our data are consistent with studies reporting T-type Ca^2+^ channels that influence vascular tone and have properties similar to high voltage activated Ca^2+^ channels, (Navarro-Gonzalez et al., 2009). Both T- and L-type Ca^2+^ channels are expressed in rat basilar (Navarro-Gonzalez et al., 2009) and middle cerebral arteries (Kuo et al., 2008), and in each artery the CaV_3.2_ (or T-type) is the most abundant VGCC alpha subunit expressed. Human recombinant T-type Ca^2+^ channels (Kaku et al., 2003) and T-type Ca^2+^ channels co-expressed with auxiliary subunits (Wyatt et al., 1998) do have gating properties similar to high voltage activated channels, so channels in the middle cerebral artery may be similar. Peripheral arteries also contain VGCCs with similar biophysical properties to high voltage activated channels (−50 to −20 mV), and are pharmacologically indistinguishable from T-type Ca^2+^ channels in both the guinea-pig and rat (Morita et al., 1999; Morita et al., 2002). In these small mesenteric arteries, T-type Ca^2+^ channels are the predominant voltage-gated subtype (Gustafsson et al., 2001; Jensen et al., 2004), and show increased window current due to non-inactivation at physiological E_m_ (Jensen and Holstein-Rathlou, 2009).

As nifedipine abolished vasomotion, we propose that L-type channels are key for the initial depolarization and constriction (Yuill et al., 2010); whereas T-type are activated subsequently and as such is critical for the vasospastic vasomotion. Interestingly, input from T-type Ca^2+^ channels seems to be important for the initial constriction in the basilar artery, whereas L-type channels are critical for vasomotion (Navarro-Gonzalez et al., 2009). But taken together, these results all suggest a functional coupling between L- and T-type Ca^2+^ channels, as previously suggested in renal (Hansen et al., 2001) and mesenteric arterioles (Braunstein et al., 2008). As such, this might explain why neuroprotection in ischemic stroke is more effective in patients given blockers for more than just L-type VGCCs (Kobayashi and Mori, 1998).

As VGCC and hence vasospastic vasomotion are inhibited by a complex interaction between NO and smooth muscle cell BK_Ca_ channels in middle cerebral arteries, we attempted to characterize further the vasospastic vasomotion. Endothelial cell damage did not affect the vasomotion, so we inhibited a variety of K^+^ currents. Both inwardly rectifying and voltage-gated K^+^ channels participate in maintenance of resting membrane potential and vascular tone (Ko et al., 2008; Nelson and Quayle, 1995 Sobey, 2001). However, inhibition of K_IR_ channels with CsCl or barium did not affect vascular tone, although CsCl did slightly increase E_m_ oscillation amplitude. As Ba^2+^ was without effect, this small change most likely reflected a non-selective action of CsCl. Inhibition of K_v_ channels with 4-AP also had little effect, causing only a small increase in the amplitude of oscillations in E_m_ and tension. This is consistent with the reported role of these channels in rat mesenteric artery where inhibition of K_v_ increased rhythmic contractions (Gustafsson and Nilsson, 1994). So voltage-gated K^+^ channels did not appear to play any major role in vasospastic vasomotion.

Our data do suggest that Na^+^/K^+^-ATPase might contribute to vasomotion, as ouabain caused relaxation and reduced the amplitude and frequency of oscillations in E_m_, although surprisingly without affecting oscillations in tension. Ouabain can attenuate intercellular communication in smooth muscle (Harris et al., 2000; Martin et al., 2003; Matchkov et al., 2007) and the synchronized changes in vascular [Ca^2+^]_i_ (Koenigsberger et al., 2004) that lead to vasomotion (Chaytor et al., 1997; Matchkov et al., 2004; Peng et al., 2001). So in part, ouabain may alter membrane potential oscillations by modifying cell–cell communication. Interestingly, ouabain effects were reversed by 4-AP, again indicating that K_V_ might contribute under some conditions to influence vasomotion.

The ability of the K_Ca_3.1 channel inhibitor TRAM-34 to reduce rather than enhance the amplitude of oscillations in E_m_ and tension was also unexpected. This effect was on the smooth muscle, as it was not altered by removal of the endothelium, and in contrast to TRAM-34, charybdotoxin, a mixed BK_Ca_ and K_Ca_3.1 inhibitor failed to modify the oscillations. One explanation is that TRAM-34 inhibits non-selective cation channels in the smooth muscle, similar to its action in isolated immune cells (Schilling and Eder, 2007). Non-selective cation channels are present in rat middle cerebral artery smooth muscle cells and appear to contribute to the calcium entry and vascular tone (Marrelli et al., 2007; Welsh et al., 2002). So data with TRAM-34 suggest that non-selective cation channels may play an important role in the calcium entry events underpinning depolarization and vasomotion after NOS inhibition in the middle cerebral artery.

In summary, inhibition of either BK_Ca_ channels and/or NOS evokes vasospasm and fast, rhythmic oscillations in E_m_ and tension that are mediated by Ca^2+^ influx via both T-type and L-type Ca^2+^ channels. Our data suggest that the T-type channels are active at physiologically relevant membrane potentials and can therefore make an important contribution to the control of cerebrovascular blood flow during vasospasm associated with disease states where NO synthesis or action is impaired, such as cerebral ischemia or subarachnoid haemorrhage.
